# Genomics of drug sensitivity in bladder cancer: an integrated resource for pharmacogenomic analysis in bladder cancer

**DOI:** 10.1186/s12920-018-0406-2

**Published:** 2018-10-03

**Authors:** Adnan Ahmad Ansari, Inkeun Park, Inki Kim, Sojung Park, Sung-Min Ahn, Jae-lyun Lee

**Affiliations:** 1 Department of Biomedical Engineering, University of Ulsan College of Medicine, Seoul, South Korea; 2 Asan Center for Cancer Genome Discovery, Asan Institute for Life Sciences, Asan Medical Center, Seoul, South Korea; 3 Division of Medical Oncology, Department of Internal Medicine, Gachon University Gil Hospital, Gachon University, Incheon, South Korea; 4 Asan Institute for Life Sciences, Asan Medical Center, Seoul, Republic of Korea; 5 Department of Genome Medicine and Science, College of Medicine, Gachon University, Incheon, South Korea; 6 Departments of Oncology, Asan Medical Center, University of Ulsan College of Medicine, Seoul, Korea; 7 Department of Internal Medicine, Asan Medical Center, University of Ulsan College of Medicine, Seoul, Korea

**Keywords:** Bladder cancer, Database, Drug response, Pharmacogenomics, Therapeutic biomarker

## Abstract

**Background:**

Bladder cancer has numerous genomic features that are potentially actionable by targeted agents. Nevertheless, both pre-clinical and clinical research using molecular targeted agents have been very limited in bladder cancer.

**Results:**

We created the Genomics of Drug Sensitivity in Bladder Cancer (GDBC) database, an integrated database (DB) to facilitate the genomic understanding of bladder cancer in relation to drug sensitivity, in order to promote potential therapeutic applications of targeted agents in bladder cancer treatment. The GDBC database contains two separate datasets: 1) in-house drug sensitivity data, in which 13 targeted agents were tested against 10 bladder cancer cell lines; 2) data extracted and integrated from public databases, including the Cancer Therapeutics Research Portal, Cancer Cell Line Encyclopedia, Genomics of Drug Sensitivity in Cancer, Kyoto Encyclopedia of Genes and Genomes, and the Cancer Gene Census databases, as well as bladder cancer genomics data and synthetic lethality/synthetic dosage lethality connections.

**Conclusions:**

GDBC is an integrated DB of genomics and drug sensitivity data with a specific focus on bladder cancer. With a user-friendly web-interface, GDBC helps users generate genomics-based hypotheses that can be tested experimentally using drugs and cell lines included in GDBC.

**Electronic supplementary material:**

The online version of this article (10.1186/s12920-018-0406-2) contains supplementary material, which is available to authorized users.

## Background

Bladder cancer is the sixth most commonly diagnosed malignancy in men [1]. Non-muscle invasive bladder cancer is associated with a good prognosis, whereas muscle-invasive or metastatic bladder cancer has a poor prognosis [2]. For metastatic bladder cancer, cisplatin-based cytotoxic chemotherapy is used as the standard first-line treatment [3]. If this fails, there is no globally accepted second-line treatment option. Recently, Bellmunt and colleagues [4] demonstrated the superior clinical performance of pembrolizumab, an immune checkpoint inhibitor, establishing pembrolizumab as the standard second-line treatment for metastatic bladder cancer.

After large-scale cancer genomics studies, scientists have developed a multitude of targeted agents based on key newly identified genomic aberrations [5]. This approach has been very successful in cancers such as melanoma, non-small cell lung cancer, and breast cancer [6]. Recent genomic studies regarding bladder cancer have demonstrated that this malignancy has numerous genomic features that are potentially actionable using targeted agents [7–9]. In fact, 56–69% of genomic aberrations in bladder cancer are associated with potentially actionable signaling pathways, such as PI3K/AKT/mTOR, RTK/MAPK, and G1-S cell cycle progression. With the exception of a small number of clinical trials, however, targeted agents have not been widely used to treat bladder cancer.

In this study, we created the Genomics of Drug Sensitivity in Bladder Cancer (GDBC) database, an integrated database to facilitate the genomic understanding of bladder cancer in relation to drug sensitivity, and thus to promote potential therapeutic applications of targeted agents to bladder cancer (http://gdbc.ewostech.net).

## Construction and content

### Data collection and processing

**Fig. 1:**
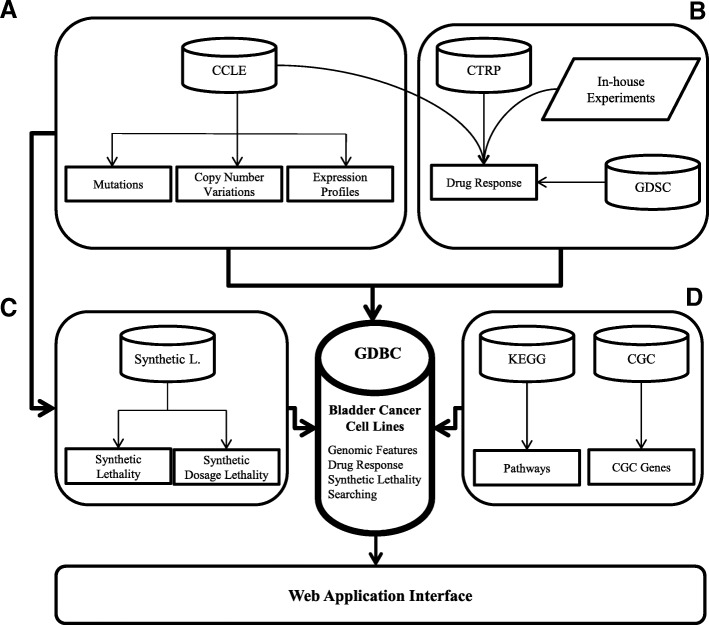
The schematic representation of GDBC. GDBC consists of two parts: 1) in-house drug sensitivity data; 2) data extracted from public databases. **a** The genomic features of bladder cancer were extracted from CCLE and the literature. **b** The drug sensitivity data were partly extracted from CTRP, GDSC and CCLE and were partly generated in-house using 13 targeted agents against 10 bladder cancer cell lines. **c** SL and SDL connections were calculated using the genomic features of bladder cancer cell lines (refer to the Methods section). **d** Pathway and cancer gene data were extracted from the KEGG and Cancer Gene Census, respectively. A web interface was developed for user-friendly access to GDBC

GDBC contains two separate datasets: 1) in-house drug sensitivity data; and 2) data extracted from public databases of 27 bladder cancer cell lines (Table 1). As for the in-house data generation, we performed drug sensitivity tests using 13 targeted agents against 10 bladder cancer cell lines (Table 2). For public data, we extracted and integrated publicly available data on 27 bladder cancer cell lines from the following data portals: 1) Cancer Therapeutics Research Portal (CTRP) [10]; 2) Cancer Cell Line Encyclopedia (CCLE) [11]; 3) Genomics of Drug Sensitivity in Cancer (GDSC) [12]; 4) Kyoto Encyclopedia of Genes and Genomes (KEGG) [13]; 5) Cancer Gene Census (CGC) [14]; 6) bladder cancer genomics data [15]; and 7) synthetic lethality (SL)/synthetic dosage lethality (SDL) connections [16]. Figure 1 illustrates how GDBC was constructed; Table 1 summarizes the pharmacogenomic landscape of 27 bladder cancer cell lines.

**Table 1:** The pharmacogenomic landscape of 27 bladder cancer cell lines

Cell Lines	Genomic Features					Drug Sensitivity			
	Mut	Del	Amp	Up-reg	Down-reg	CTRP	GDSC	CCLE	GDBC
5637	1400	117	85	1123	1404	463	98	24	10
639 V	299	135	203	1430	1235	467	99	24	0
647 V	96	342	218	1394	1143	451	99	0	0
BC3C	NA	158	37	1298	1277	468	0	0	0
BFTC905	92	549	312	1201	1394	357	99	0	0
CAL29	73	119	637	1151	1420	404	0	0	0
HS172T	47	62	9	1817	1733	0	0	0	0
HT1197	744	113	266	1898	1498	443	99	24	8
HT1376	775	242	455	1583	1614	465	99	24	7
J82	760	324	135	1166	1191	470	95	22	9
JMSU1	96	272	121	1620	1332	463	0	24	0
KMBC2	121	320	122	1496	1616	474	0	24	0
KU1919	91	124	354	1164	1330	461	99	0	0
RT112	903	889	133	1466	1348	442	99	24	0
RT11284	72	163	20	1371	1177	0	0	0	0
RT4	1094	115	30	1458	1384	458	98	24	10
SCABER	85	144	173	1224	1306	455	0	24	0
SW1710	772	161	35	1366	1334	384	99	0	0
SW780	895	187	30	1319	1455	0	99	0	11
T24	NA	121	7	1315	982	473	99	24	8
TCCSUP	720	136	207	2040	1685	467	99	24	0
UBLC1	73	NA	NA	1232	1591	467	0	0	0
UMUC1	79	217	331	1928	1591	449	0	0	0
UMUC3	879	175	83	1456	1063	465	95	24	10
VMCUB1	959	128	160	1428	1586	468	99	0	0
253 J	1624	NA	NA	1406	1569	410	0	0	4
253 JBV	72	NA	NA	1187	1392	377	0	0	12

**Table 2:** IC50 values of 13 targeted agents in 10 bladder cancer cell lines, with their molecular targets indicated

Drug	Target	HT1376	J82	RT4	T24	UMUC3	5637	SW780	253 J	253 JBV	HT1197
Afatinib	EGFR: HER2	NA	3.93	3.08	4.41	4.54	0.43	3.7	1.85	1.53	NA
Axitinib	PDGFR: KIT: VEGFR	8.95	> 10	7.34	9.25	6.44	3.15	14.5	NA	> 10	> 10
Caborazantinib	MET: RET: VEGFR2	9.55	> 10	2.93	NA	> 10	9.22	6.53	NA	> 10	> 10
Erlotinib	EGFR	5.62	NA	6.9	7.99	> 10	3.41	> 10	NA	> 10	> 10
Everolimus	mTOR	3.77	2	> 10	0.33	0.67	2.2	> 10	NA	1.5	0.71
GDC-0879	RAF	NA	NA	NA	NA	NA	NA	NA	NA	> 10	NA
Lapatinib	EGFR:ERBB2	2.73	5.12	0.57	6.82	2.49	1.66	1.45	NA	2.35	4.7
Lonafanib	FNTB	NA	> 10	> 10	> 10	> 10	> 10	> 10	> 10	> 10	NA
Nutlin-3	p53-MDM2 interaction	NA	NA	NA	NA	NA	NA	> 10	NA	> 10	> 10
Gefitinib	AKT1:EGFR	NA	> 10	> 10	> 10	> 10	1.14	NA	3.11	3.8	NA
Trametinib	MEK	NA	NA	2.54	NA	NA	NA	> 10	> 10	NA	NA
Vermurafenib	BRAF	> 10	> 10	NA	NA	> 10	> 10	> 10	NA	> 10	> 10
Vorinostat	HDAC inhibitors Class I, IIa, IIb, IV	2.48	2.95	1.52	0.9	2.35	1.2	1.36	NA	1.07	3.47

The value represents IC50 (μM)

### In-house drug sensitivity experiments

We performed drug sensitivity experiments using 13 targeted agents against 10 bladder cancer cell lines (Table 2). These 13 targeted agents were selected based on the potential actionable genomic aberrations identified through bladder cancer genomic analyses [7–9].

All cells were maintained as per the recommendations of the ATCC or other references. Briefly, 1 day before the treatment, cells were plated at a density of 3–5 × 10^3^ cells/well in 80 μL of culture media within a 96-well plate. After overnight incubation, cells were treated with each drug at the indicated concentration in 20 mL of culture medium. To calculate the IC50 of the test drug, cells were treated with 10 serially diluted concentrations of drugs (25% serial dilution) from a highest concentration of 10 μM (10 μM, 2.5 μM, 0.625 μM, 0.015625 μM, 0.0039 μM, 0.000975 μM, 0.00024375 μM, 0.0000609 μM, 0.0000152 μM, 0.0000038 μM), and after 48 h of incubation, cellular adenine-triphosphate (ATP) content was evaluated using the CellTiterGlo assay (Promega). Every dilution step was performed to maintain same concentration of DMSO. The raw ATP values were recorded and transferred to Prism software. Raw values from non-treated control (non-treated control implies DMSO treated with same percentage in culture media) wells were adjusted to 100% of survival, and the relative survival rates were calculated by dividing the ATP luminescence values of the test well by those of the control wells. IC50 values of each drug in different cells were calculated by performing non-linear regression analysis. Data represent the mean values of calculated IC50 from two independent experiments.

### Data extraction

Raw datasets from CTRP [17], CCLE [18] and GDSC [19] were downloaded. Previously published mutation data from the literature were extracted and added to the CCLE and GDSC data to enhance the information [15]. The cancer gene census (CGC) genes are also stored in a database through which cancer researchers can easily identify hotspot genes. SL/SDL connections were taken from Jerby-Arnon et al. and the *p*-value of each connection was recalculated using only bladder cancer cell lines [16]. The method of p-value calculation is available on a website (http://gdbc.ewostech.net/Documentation.php); we modified the original algorithm because of data limitations. Finally, we stored all this information in a database for easy access. Furthermore, information was downloaded from KEGG and stored in a database that helps the user to identify the importance of a particular gene by looking at its pathways.

### Parameters for the CNV, expression and drug sensitivity analysis

The normalized CNV values were downloaded from CCLE website and stored in a database [18]. We considered values greater than 1 to be amplifications and lower than 1 to be deletions. Amplifications are shown in red and deletions are shown in blue.

Our method is based on Jerby-Arnon’s method that was used for the CCLE database [16]. In cancer genomic studies, differential expression analysis is usually performed by comparing the gene expression values of tumor samples with those of matched normal samples. In the case of cell lines (e.g., as in CCLE), however, there are no normal counterparts to be compared. Jerby-Arnon et al. used percentile based method to detect up-regulation and down-regulation in CCLE cell lines. In this study we scored up-regulation and down-regulation based on the following two conditions: 1) The values should differ at least by 15% from the mean of all other available cell lines in CCLE irrespective of cancer type; 2) We calculated the percentiles using all cell lines in CCLE and it should be the top 10 percentile of genes in CCLE were classed as up-regulated and the bottom 10 percentile as down-regulated. The calculated expression shows the difference of expression from the average of the remaining cell lines; 100% is exactly the same as the average. Up-regulations are shown in red and down-regulations are shown in blue.

Drug sensitivity for GDSC and CCLE is decided on the basis of the IC50 value: if the IC50 value is below 1 the block will be in red, showing high sensitivity, but users can freely decide sensitivity by comparing the IC50 values of that drug in other cell lines. GDSC has one of the largest drug sensitivity datasets across cancer cell lines. We extracted bladder cancer drug-related data from GDSC, which are comprised of data obtained from 224 different drugs tested against different bladder cancer cell lines. The average IC50 is the average sensitivity of those drugs in different available cell lines across whole database. Sensitive cell lines are shown in red. The graph shows the IC50 values of different drugs in comparison with the average IC50 and other selected cell lines. Furthermore, CCLE contains a dataset of 24 clinically relevant drugs and data of 13 clinically relevant drugs that was tested in-house against 10 cell lines. For CTRP, ~ 475 compounds were tested against bladder cancer cell lines and the subsequently generated area under curve (AUC) from all these lines is available to download. The AUC values are converted using the R extreme values software package and outliers are considered as sensitive or resistant. All these datasets are part of GDBC.

### SL and SDL connections

Synthetic lethality (SL) is when one gene is inactive and another gene is essential for cell survival. On the other hand, synthetic dosage lethality (SDL) is when one gene is over-active and another gene is essential for cell survival. The SL and SDL algorithm was adapted from Jerby-Arnon et al. with some modifications because of data limitations [16]. We removed the shRNA aspect from the algorithm; complete details of the algorithm and the parameters of the calculations are available on the website under the documentation section.

### Database and website development

All data are stored in a MySQL database (v. 5.5.47) after processing. All mutations, CNV and expression data are converted into tabular form for storage in the database. The website was developed in PHP5 and deployed on an Apache2 server.

### The pharmacogenomic annotation of bladder cancer cell lines

GDBC contains pharmacogenomic data of 27 bladder cancer cell lines. Table 1 summarizes the data included in GDBC for each of the 27 bladder cancer cell lines. For example, bladder cancer cell line 5637 has 1400 mutations, 117 gene deletions, 85 gene amplifications, 1123 gene up-regulations and 1404 gene down-regulations. In addition to the genomic information, GDBC provides drug response data for the 5637 cell lines against 463 compounds from CTRP, 217 compounds from GDSC, 24 compounds from CCLE, and 10 compounds tested in-house.

### Comparison of genomic features between bladder cancer cell lines and tumors

It has been questioned whether cancer cell lines are true representatives of real cancers. We performed a systematic comparison of genomic profiles from bladder cancer tissues [7] and those of bladder cancer cell lines present in GDBC. First, at the gene expression level, bladder cancer cell lines showed gene expression patterns that were similar to those of bladder cancer tissues. For example, *APOBEC3B*, *EGFR*, *KRT14*, *KRT5*, *KRT6A* and *AKT3* were up-regulated in both datasets. Second, at the DNA level, bladder cancer cell lines harbored the majority of functionally important CNVs and mutations identified in bladder cancer tissues. For example, frequent deletions in *CDKN2A*, *PDE4D*, *RB1*, *FHIT*, *FAM190A*, *LRP1B*, and *WWOX* and amplifications in *E2F3*, *CCND1*, *PPARG*, and *EGFR* were observed in both datasets. Furthermore, *TP53*, *FGFR3*, *PIK3CA*, *TSC1*, *RB1*, *KDM6A*, *CREBBP*, *EP300*, and *ARID1A* were frequently mutated in both datasets [20]. In summary, bladder cancer cell lines had many of the potentially actionable genomic features identified in bladder cancer tissues and thus appear to be suitable for pharmacogenomic studies (Additional file 1: Figure S1) [21].

## Utility and discussion

### A user-friendly web interface

When developing GDBC, we assumed that the main users of GDBC would be cancer biologists and clinicians involved in bladder cancer research. Using the web interface, researchers can extract meaningful information from GDBC in multiple ways by using simple keywords as search terms. Two use case scenarios of GDBC are described below.

### Inhibition of the fibroblast growth factor receptor (FGFR) pathway

#### Research question

The fibroblast growth factor/fibroblast growth factor receptor (FGF/FGFR) is a receptor tyrosine kinase (RTK) signaling pathway that plays important roles in diverse cell functions, including proliferation, differentiation, apoptosis and migration [22]. The dysregulation of *FGFR1* and *FGFR3* is common in bladder cancer. Additionally, FGFR inhibitors are under clinical investigation in other cancer types. For example, AZD4547, a selective FGFR (FGFR 1–3) inhibitor, inhibited cell proliferation in both cancer cell lines and tumor xenograft models, in which the FGFR pathway was activated [23]. PD173074, a pan-FGFR inhibitor, blocked the growth of small cell lung cancer (SCLC) both in vitro and in vivo [24]. Based on these backgrounds, we questioned whether there would be any pharmacogenomic relationship between *FGFR1*/*FGFR3* dysregulation and FGFR inhibitors in bladder cancer.

#### GDBC interrogation

To address this question, we first performed a gene-centric search; we simply typed *FGFR1* and *FGFR3* into the gene search box to search for these genomic features in GDBC. According to the gene search results of GDBC, *FGFR1* and *FGFR3* were up-regulated in ~ 15% and ~ 20% bladder cancer cell lines, respectively. In addition, *FGFR3* was non-synonymously mutated in ~ 11% of bladder cancer cell lines. The gene-centric search result also provided information on three drugs (namely, AZD4547, PD-173074 and nintedanib) that target both *FGFR1* and *FGFR3*.

Then, we performed a drug-centric search using the drug information acquired from the gene-centric search. According to the drug search results, AZD4547 was tested in 23 bladder cancer cell lines; three cell lines (RT112, JMSU1, and UMUC1) were sensitive to AZD4547. PD-173074 was tested in 16 bladder cancer cell lines; RT112 was remarkably sensitive to PD-173074, whereas RT4, SW780, and 639 V showed a partial response to PD-173074. Nintedanib was tested in 20 bladder cancer cell lines; 639 V and KMBC2 showed only a slight response to Nintedanib. In RT112, which was responsive to both AZD4547 and PD-173074, *FGFR3* was substantially up-regulated. In JMSU1 and UMUC1, which were both responsive to AZD4547, *FGFR1* and *FGFR3* were each up-regulated, respectively. Altogether, these findings suggest that it is worth experimentally testing the pharmacogenomic relationship between the dysregulation of *FGFR1* and *FGFR3* and FGFR1/FGFR3 inhibitors in bladder cancer.

### Expression of EGFR and sensitivity to EGFR inhibitors

#### Research question

The epidermal growth factor receptor (*EGFR*) is a key factor in epithelial malignancies, and its activity enhances tumor growth, invasion, and metastasis [25]. *EGFR* is highly expressed in several cancers, and is a critical factor in driving tumorigenesis. Various drugs targeting *EGFR* (i.e., erlotinib, lapatinib, gefitinib, afatinib, etc.) have been approved for the treatment of several cancers [26]. EGFR is up-regulated in ~ 19% of bladder cancer cases [7], being a potentially actionable target for therapeutic manipulation in bladder cancer. Based on this background, we questioned whether there would be any pharmacogenomic relationship between EGFR up-regulation and drugs targeting ERBB family members in bladder cancer.

#### GDBC interrogation

**Fig. 2:**
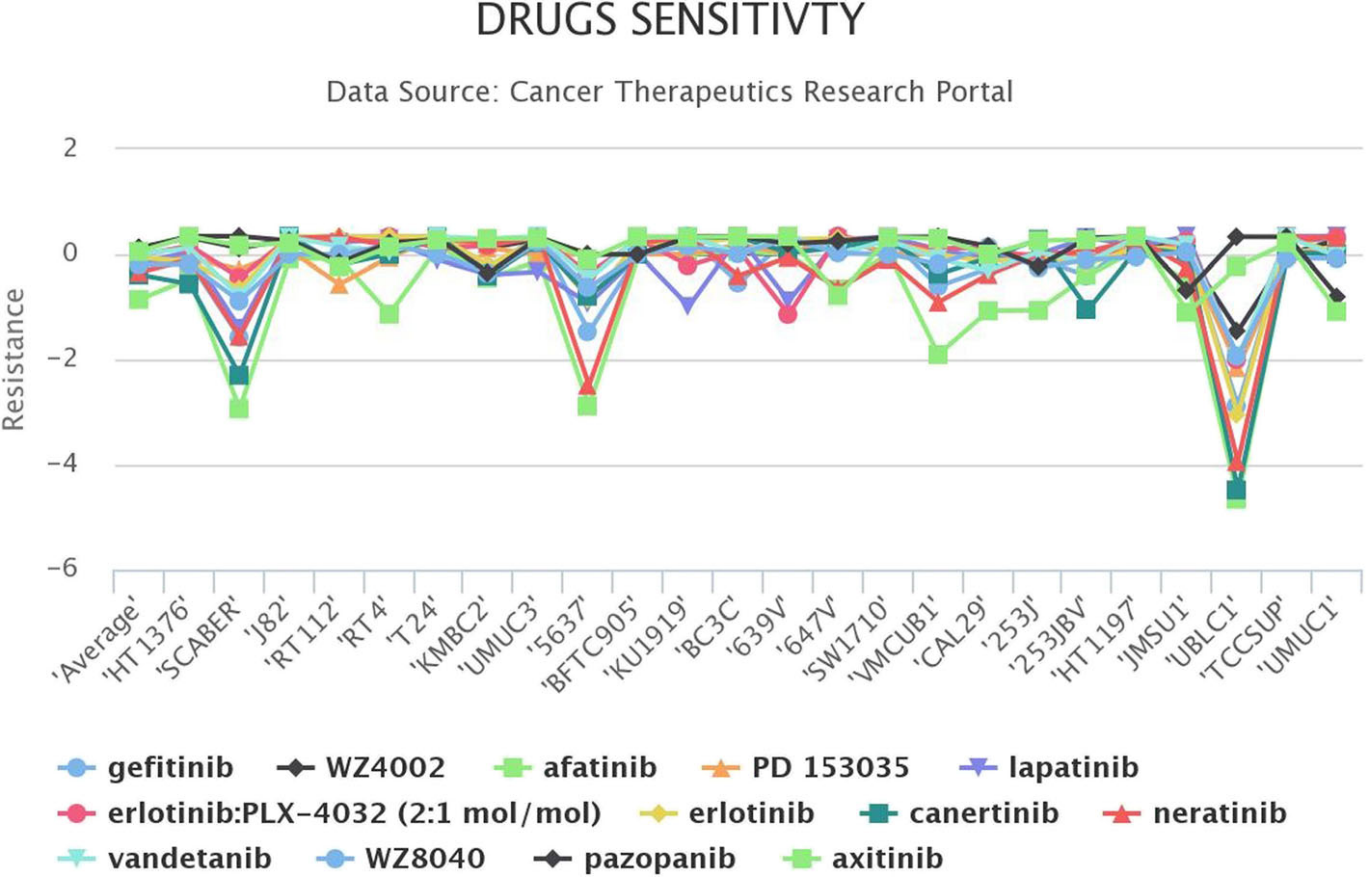
Drug sensitivity of EGFR inhibitors in bladder cancer cell lines. Bladder cancer cell lines with high expression of EGFR, including HT1376, 5637 and UBLC1, were markedly sensitive to EGFR-targeting agents. The average IC50 is the average sensitivity of those drugs in different available cell lines across CTRP

To answer this question, we first performed a gene-centric search by typing *EGFR* into the gene search box. This simple search revealed that *EGFR* was significantly up-regulated in ~ 18% of bladder cancer cell lines and that a number of EGFR-targeting drugs (i.e., gefitinib, WZ4002, afatinib, PD 153035, lapatinib, erlotinib, canertinib, neratinib, vandetanib, WZ8040, Pazopanib, Axitinib, AMG-706, BIBW2992, ZD-6474, and Caborazantinib) were tested in bladder cancer cell lines. Then we performed a drug-centric search using the drug information acquired from the gene-centric search. According to our drug search results, bladder cancer cell lines with relatively high expression of *EGFR* were more sensitive to EGFR-targeting agents (Fig. 2). Bladder cancer cell lines with high expression of *EGFR*, including SCABER, 5637 and UBLC1, were markedly sensitive to EGFR-targeting agents. Altogether, these findings suggest that it is worth experimentally testing the pharmacogenomic relationship between the *EGFR* up-regulation and EGFR-targeting agents in bladder cancer.

## Discussion

This study was motivated by two key factors: 1) despite numerous genomic features that are potentially actionable by targeted agents, both pre-clinical and clinical studies using molecular targeted agents have been very limited in bladder cancer; 2) public databases have their own strengths and weaknesses in terms of genomics and drug sensitivity data. Furthermore, drug sensitivity data for bladder cancer cell lines are very limited.

In this study, we have created GDBC, an integrated database to facilitate the genomic understanding of bladder cancer in relation to drug sensitivity, and thus to promote the potential therapeutic applications of targeted agents to bladder cancer. GDBC includes data not only from public databases such as CTRP, CCLE, and GDSC, but also from in-house experiments specifically targeted against bladder cancer. CTRP, CCLE, and GDSC are three major pharmacogenomics DBs with different strengths and weaknesses; CCLE has genomic information with very limited drug information; GDSC has drug information with very limited genomic information; and CTRP has other limitations because of a very stringent interface (i.e., one cannot query against a particular cell line or tissue of origin). Our main goal for this study was to combine all bladder cancer-related public and in-house data in one platform with a user-friendly interface.

Implementation of precision cancer medicine requires in vitro and in vivo proof-of-concept studies followed by clinical trials. GDBC is a powerful tool in that researchers can easily identify potential pharmacogenomic relationships in bladder cancer. Based on the hypotheses generated by GDBC interrogation, researchers can perform further in vitro and in vivo validation and, eventually, clinical studies.

As mentioned above, GDBC contains both in-house and public data. One of the typical drawbacks in integrating different data sources is the potential inconsistency of the data. These types of inconsistencies may result from a number of biological and methodological factors that differ between the data sources. For example, the number of cells seeded per well, the drug concentration range examined, the number of cell doublings achieved, the types of cell viability assay, the analytical tools to calculate drug sensitivity, and so on [27]. In short, this type of inconsistency is one of the inevitable features of data integration. Therefore, when encountering any inconsistency, researchers need to investigate experimental details that may have caused the inconsistency between different data sources and to identify a way to validate pharmacogenomic relationships in their own contexts.

In order to provide users with updated information, we have developed a computational pipeline to update the GDBC database. Currently, our plan is to automatically update GDBC every 3 months and manually on any requests. Users can share their data with us in simple tab delimited text files that will be parsed by our pipeline and will be added into the GDBC database.

## Conclusions

GDBC is an integrated pharmacogenomic database specialized for bladder cancer. GDBC can be used as a tool to facilitate the genomic understanding of bladder cancer in relation to drug sensitivity, and thus to promote potential therapeutic applications of targeted agents to bladder cancer.

## Additional file

Additional file 1: Figure S1. Heat map of frequent genetic aberrations in bladder cancer patient samples from cBioPortal demonstrates that genetic aberrations found in bladder cancer cell lines are compatible to those found in bladder cancer tissue samples; the left side shows the bladder cancer patient tissue samples and the right side shows the bladder cancer cell lines. A) Frequently mutated genes in bladder cancer. B) Frequently upregulated genes in bladder cancer. C) Frequently deleted genes in bladder cancer. D) Frequently amplified genes in bladder cancer. The frequencies of some genes are not comparable between bladder cancer tissue data and bladder cancer cell line data. We believe there are at least two reasons: 1) 27 bladder cancer cell lines may not cover the full mutational spectrum of bladder cancer, especially relatively rare mutations; 2) There may be some technical issues related to differential gene expression analyses. As for tissue data, usually both cancer and normal tissue data are available, and we detect differentially expressed genes by comparing these paired data sets. As for cell line data, this pairwise comparison cannot be done, and therefore, cell line data are handled quite differently for differential gene expression analyses as we described in the Method section. The difference of data handling in cancer tissue and cell lines may cause some discrepancy in detecting differentially expressed genes. (PNG 133 kb)

## Abbreviations

AUC: Area under curve
CCLE: Cancer Cell Line Encyclopedia
CGC: Cancer Gene Census
CTRP: Cancer Therapeutics Research Portal
GDBC: Genomics of Drug Sensitivity in Bladder Cancer
GDSC: Genomics of Drug Sensitivity in Cancer
KEGG: Kyoto Encyclopedia of Genes and Genomes
SDL: Synthetic dosage lethality
SL: Synthetic lethality
