# Supplementary figures and images for: Genomics of drug sensitivity in bladder cancer: an integrated resource for pharmacogenomic analysis in bladder cancer
Source: BMC Med Genomics. 2018 Oct 3;11:88. doi: 10.1186/s12920-018-0406-2 (PMC6171176; doi:10.1186/s12920-018-0406-2)

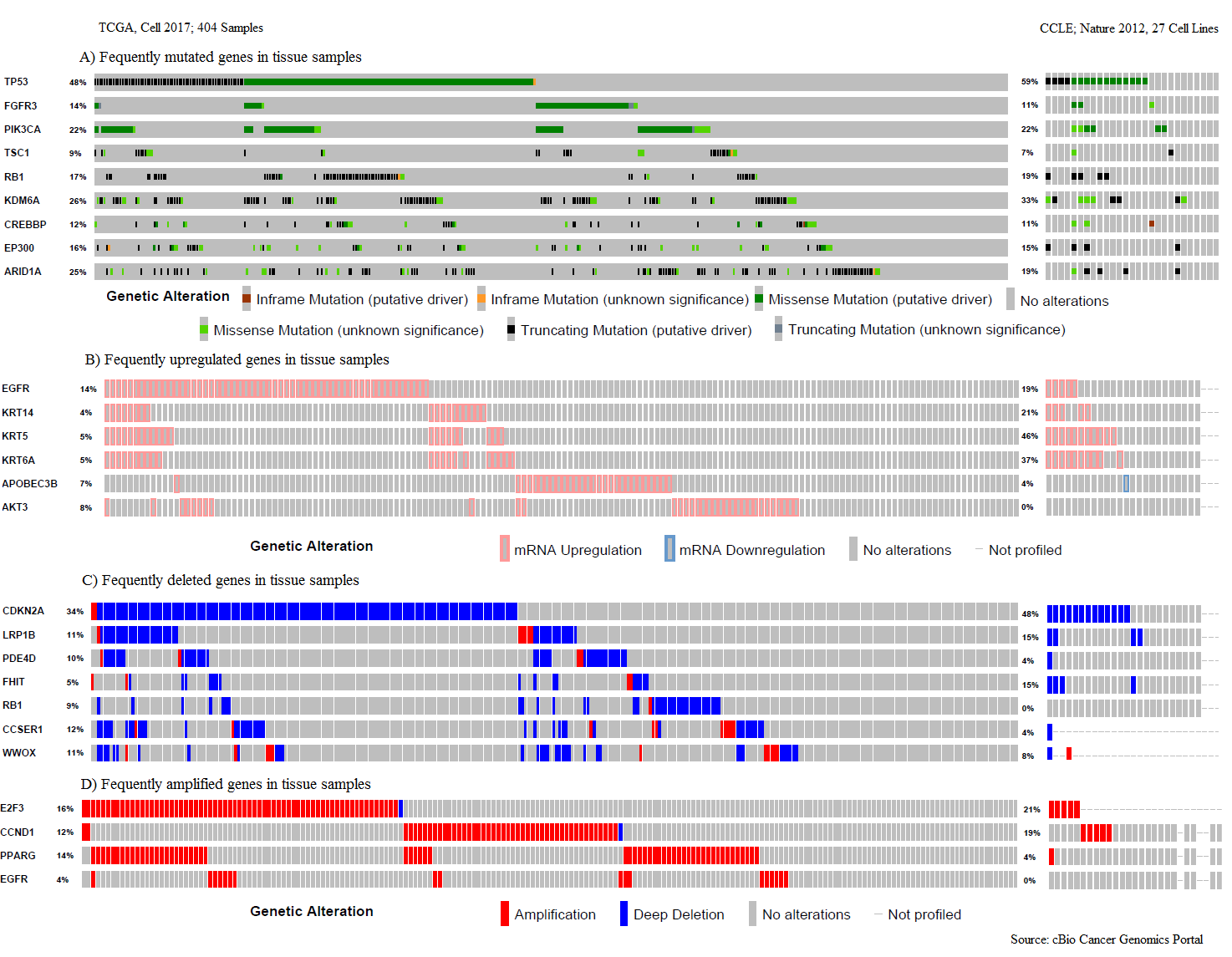

Supplement: Supplementary file 1 — Figure S1. Heat map of frequent genetic aberrations in bladder cancer patient samples from cBioPortal demonstrates that genetic aberrations found in bladder cancer cell lines are compatible to those found in bladder cancer tissue samples; the left side shows the bladder cancer patient tissue samples and the right side shows the bladder cancer cell lines. A) Frequently mutated genes in bladder cancer. B) Frequently upregulated genes in bladder cancer. C) Frequently deleted genes in bladder cancer. D) Frequently amplified genes in bladder cancer. The frequencies of some genes are not comparable between bladder cancer tissue data and bladder cancer cell line data. We believe there are at least two reasons: 1) 27 bladder cancer cell lines may not cover the full mutational spectrum of bladder cancer, especially relatively rare mutations; 2) There may be some technical issues related to differential gene expression analyses. As for tissue data, usually both cancer and normal tissue data are available, and we detect differentially expressed genes by comparing these paired data sets. As for cell line data, this pairwise comparison cannot be done, and therefore, cell line data are handled quite differently for differential gene expression analyses as we described in the Method section. The difference of data handling in cancer tissue and cell lines may cause some discrepancy in detecting differentially expressed genes. (PNG 133 kb) [file 12920_2018_406_MOESM1_ESM.png]
